# Effects of Tezepelumab in “United Airway Disease” (Asthma and CRSwNP)

**DOI:** 10.1155/crpu/6616379

**Published:** 2026-03-03

**Authors:** Chiara Lupia, Giovanna Lucia Piazzetta, Daniela Pastore, Nadia Lobello, Mariaimmacolata Preianò, Emanuela Chiarella, Angelantonio Maglio, Alessandro Vatrella, Girolamo Pelaia, Corrado Pelaia

**Affiliations:** ^1^ Department of Health Sciences, “Magna Græcia” University, Catanzaro, Italy, unicz.it; ^2^ Department of Medical and Surgical Sciences, “Magna Græcia” University, Catanzaro, Italy, unicz.it; ^3^ Department of Medicine, Surgery and Dentistry, University of Salerno, Salerno, Italy, unisa.it

**Keywords:** nasal polyps, severe asthma, T2-low, tezepelumab, TSLP

## Abstract

Severe asthma is a heterogeneous disease typically characterized by inadequate symptom control, even with frequent oral corticosteroids, large doses of inhaled corticosteroids, and long‐acting *β*_2_‐adrenergic agonists. The latest Global Initiative for Asthma (GINA) recommendations suggest adding biological therapies at Step 5 to optimize standard treatments. Although there is a wide range of therapeutic options for T2‐high asthma, unfortunately, for T2‐low asthma, we still have limited therapeutic choices. Our case report refers to a 64‐year‐old woman with severe T2‐low asthma and chronic rhinosinusitis with nasal polyps (CRSwNP) who had already tested other standard therapies without clinical or functional benefits. She is being treated with tezepelumab (210 mg subcutaneous injection, administered every 4 weeks), an antithymic stromal lymphopoietin (TSLP) human monoclonal antibody. After the third dose, she showed significant clinical and functional benefits, which were also confirmed after 6 months of add‐on biological therapy with anti‐TSLP. In conclusion, this case study suggests that tezepelumab can provide rapid and effective therapeutic action in patients with severe T2‐low asthma and nasal polyposis.

## 1. Background

Asthma is a common disease affecting approximately 300 million individuals. It is characterized by chest issues such as coughing, difficulty breathing, wheezing, and tightness, typically associated with fluctuating airflow restrictions. A small percentage of patients, around 5%–10%, experience severe uncontrolled asthma, which contributes significantly to medical, social, and economic challenges.

Regarding severe asthma, the latest Global Initiative for Asthma (GINA) recommendations suggest that biologic therapies should be added in Step 5 to optimize standard treatments [1].

Lately, tezepelumab has been approved for the treatment of severe asthma. This monoclonal antibody specifically inhibits thymic stromal lymphopoietin (TSLP), which plays an essential role in the T2‐high and T2‐low pathobiology of asthma [2]. The use of TSLP‐targeted medicines to counteract this alarmin′s effects has been the subject of several investigations. According to the PATHWAY research, tezepelumab, regardless of baseline eosinophil count, decreased clinically severe asthma exacerbations by 62%–71% compared with placebo [3]. Additionally, tezepelumab reduced the incidence of exacerbations in individuals with nasal polyps more than in patients without nasal polyps, according to a post hoc analysis of the PATHWAY research [3]. The UPSTREAM trial was a second Phase 2, double‐blind, placebo‐controlled, randomized study that concluded that independent of baseline blood eosinophil levels, a decrease in eosinophilic airway inflammation may have contributed to the improvements in clinical asthma outcomes brought about by tezepelumab in earlier investigations [4]. NAVIGATOR is a multicenter, placebo‐controlled, double‐blind, randomized trial, suggesting that by Week 52, tezepelumab, given subcutaneously at a dose of 210 mg every 4 weeks, accomplished its primary objective of reducing acute asthma exacerbations. All recruited patients exhibited this impact, including those with blood eosinophil counts below 300 and 150 cells/*μ*L [5]. DESTINATION is a long‐term extension study primarily aimed at evaluating the safety profile of tezepelumab in subjects with severe asthma over 104 weeks of therapy, including the previous treatment period [6]. The Phase 2 double‐masked, randomized, placebo‐controlled research CASCADE examines tezepelumab′s anti‐inflammatory properties. Regarding the baseline, the CASCADE trial is aimed at evaluating the potential inflammatory changes induced by biological therapy with tezepelumab [7].

The Phase III WAYPOINT study has yielded promising results in individuals with nasal polyps. Compared with a placebo, tezepelumab showed a statistically significant and clinically relevant decrease in nasal congestion and polyp size [8].

Therefore, we decided to treat a woman with T2‐low severe asthma and nasal polyps who had already undergone treatment with mepolizumab, dupilumab, and benralizumab without clinical or functional benefits with tezepelumab. In this patient, we evaluated the impact of the first six doses of tezepelumab on lung function, symptom control, serum IgE, OCS intake, asthma exacerbations, and nasal polyps. This case uniquely documents a coordinated response of the united airway to tezepelumab in a patient with severe T2‐low asthma and CRSwNP who showed no clinical benefit from mepolizumab, benralizumab, or dupilumab. Beyond confirming efficacy, we offer an integrated view of time‐matched lower‐ and upper‐airway outcomes under anti‐TSLP therapy in a real‐world setting.

## 2. Case Report

In March 2024, a 64‐year‐old woman, an ex‐smoker who had smoked 30 cigarettes per day for 40 years, came to our Respiratory Unit at “Magna Graecia” University Hospital in Catanzaro, Italy. She was diagnosed with severe asthma and nasal polyps in 2021 at another Pulmonology Center. Moreover, distinguishing T2‐low asthma from chronic obstructive pulmonary disease (COPD) or asthma‐COPD overlap (ACO) was crucial, since the patient was an ex‐smoker. In this case, the diagnosis of severe asthma with CRSwNP was supported by the onset of respiratory symptoms before the age of 40, the documented reversibility of airway obstruction on spirometry, and the absence of emphysema or COPD‐related changes on imaging and clinical evaluation. She had previously begun various biological therapies (mepolizumab, dupilumab, and benralizumab) without any significant clinical or functional benefits. External‐center datasets for prior biologic prescriptions were incomplete; therefore, detailed longitudinal trajectories under other monoclonal antibodies could not be reproduced here. Nasal polyp tissue showed epithelial remodeling with focal goblet‐cell hyperplasia and areas of squamous metaplasia, a thickened basement membrane, and a subepithelial stroma characterized by dense collagen deposition and mucous‐gland hyperplasia, with no evidence of an active, marked eosinophilic nasal mucosal infiltrate. The histopathological evaluation was purely descriptive and no cell count was performed; therefore, the percentage of eosinophils in the nasal mucosa could not be quantified.

**Figure 1:**
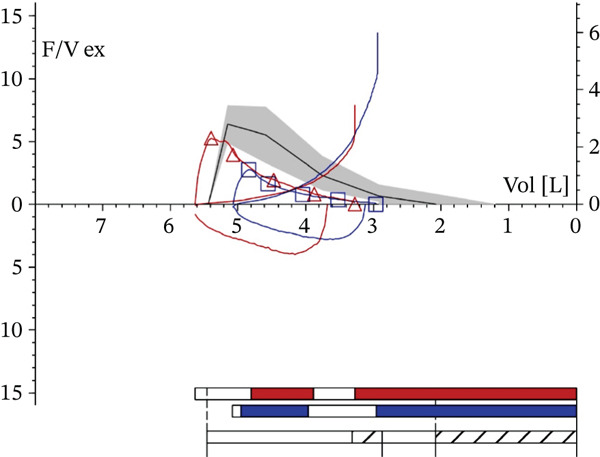
Spirometry with a positive reversibility test at baseline. “F/V ex” indicates the expiratory flow–volume curve. The vertical axis represents expiratory flow (L/s), and the horizontal axis represents lung volume (L). The bar graph in the lower right corner summarizes the maneuver timeline, pre (blue) and post (red) reversibility, with quality/repeatability summary (white).

The patient arrived at our respiratory unit complaining of coughing, difficulty breathing, wheezing, hyposmia, and nasal obstruction. At baseline, she was undergoing treatment with a single inhaler containing an extra‐fine triple treatment of beclomethasone dipropionate/formoterol fumarate/glycopyrronium (BDP/FF/G) and methylprednisolone 2 mg as needed since she had already discontinued the biological therapy with benralizumab 3 months earlier. Lung function tests were conducted on 5 March 2024. They documented a marked reversible severe obstructive ventilatory defect, with the following values: forced expiratory volume in 1 s (FEV_1_) 1.25 L (48% pred.); forced vital capacity (FVC) 2.13 L (63% pred.); Tiffenau index (FEV_1_/FVC) 58.92%; peak expiratory flow (PEF) 2.76 L/s (43% pred.); average expiratory flow over the middle half of the FVC maneuver (MMEF_25–75_) 0.70 L/s (31% pred.) (Figure 1). At baseline (T0), the result of the Asthma Control Test (ACT) was 11, the Sino‐nasal Outcome Test 22 (SNOT‐22) score was 83, and the visual analogue scale (VAS) score was 8. Physiological chest breathing sounds were diffusely reduced, and relevant wheezing was present. The skin prick test and Rast test were negative. Total serum IgE levels were 17.6 IU/mL. Blood eosinophil count was 0.2% (10 cells/*μ*L). The patient′s immunoglobulin levels for IgG, IgA, and IgM were normal, indicating no hypogammaglobulinemia or abnormalities in IgG subclasses.

Due to inadequate asthma control despite standard treatment strategies already attempted, we decided to initiate tezepelumab, with a 210 mg subcutaneous injection administered every 4 weeks. After 3 months (T3) of add‐on therapy with anti‐TSLP, we observed significant improvements in functional values (FEV_1_ 1.42 L, FVC 2.18 L, FEV_1_/FVC 65.1%, PEF 3.79 L/s, MMEF_25–75_ 0.89 L/s). Total serum IgE was 8.1 IU/mL, whereas ACT scored 17. SNOT‐22 was 62, and VAS was 6.

**Figure 2:**
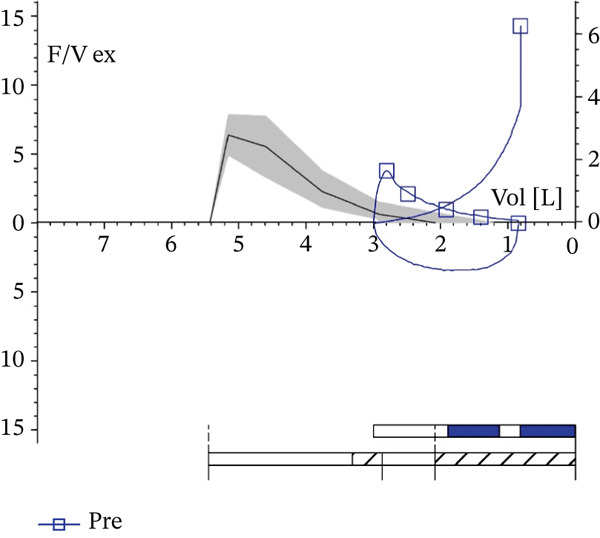
Spirometry after 6 months of add‐on biological therapy with tezepelumab. “F/V ex” indicates the expiratory flow–volume curve; “Pre” indicates prebronchodilator values. The vertical axis represents expiratory flow (L/s) and the horizontal axis represents lung volume (L). The bar graph in the lower right corner illustrates the maneuver timeline, with quality/repeatability summary (white).

Then, we gathered data after six administrations (T6), observing an FEV_1_ of 1.49 L (57% pred.), an FVC of 2.39 L (71% pred.), and a Tiffenau index (FEV_1_/FVC) of 62.6% (Figure 2). The ACT score was 21, and the total serum IgE was 7 IU/mL. Furthermore, the woman reported the lack of necessity to use a reliever as needed or OCS and to go to the hospital or medical clinics.

At this time, the patient reported a marked improvement in hyposmia and nasal obstruction. Therefore, an otorhinolaryngological visit was conducted. The rhinoscopy revealed that the nasal septum was considerably aligned with hypertrophy of the nasal turbinates, which were covered by pale mucosa, endonasal secretion, and a clear ostiomeatal complex (OMC). SNOT‐22 was 41, and VAS was 4.

## 3. Discussion

This case report is aimed at enhancing the clinical and functional effects of tezepelumab on a 64‐year‐old woman suffering from uncontrolled asthma and CRSwNP. Severe asthma shows temporal variation in both clinical phenotype and inflammatory endotype. Biomarker profiles (e.g., blood eosinophils, FeNO, and total/seasonal IgE) can change with infections, environmental exposures (including smoking), and treatment effects (high‐dose ICS, OCS bursts, and prior biologics). Therefore, classification based on a single assessment may misrepresent long‐term biology. In this case, earlier treatment choices targeting IL‐5/IL‐5R or IL‐4R*α* reflected a period when a stronger Type‐2 signal was suspected, whereas the later T2‐low profile at referral matched a favorable response to upstream TSLP inhibition, which can influence multiple downstream pathways independent of baseline eosinophil counts. Tezepelumab is a human monoclonal IgG antibody that targets a cytokine produced by epithelial cells. It works by blocking TSLP from binding to its receptor, thereby reducing the immunological response that TSLP may elicit in certain asthma endotypes. Patients with asthma exhibit higher levels of TSLP expression in their airways, which has been linked to the severity of the illness. Blocking TSLP may prevent immune cells from releasing pro‐inflammatory cytokines, thus improving asthma control and reducing flare‐ups. Operating at the top of the cascade, tezepelumab helps prevent inflammation at its source and may effectively treat many individuals with severe asthma. In this case, we demonstrated the functional impact of this new drug on spirometry values and lung inflation indices in the 64‐year‐old asthmatic woman.

We considered 6 months, from T0 (before the first injection of tezepelumab 210 mg) to T6 (after the sixth subcutaneous administration).

In fact, over the period considered in the study, FEV_1_ improved from 1.25 L (48% pred.) to 1.49 L (57% pred.), FVC from 2.13 L (63% pred.) to 2.39 L (71% pred.), Tiffenau index (FEV_1_/FVC) from 58.92% to 62.62%, PEF from 2.76 L/s (43% pred.) to 3.38 L/s (54% pred.), and MMEF_25–75_ from 0.70 L/s (31% pred.) to 0.87 L/s (39% pred.) (Table 1). Our results align with those of two multicenter, randomized, double‐masked, placebo‐controlled trials, such as PATHWAY and NAVIGATOR [3, 5]. These two studies demonstrated the functional efficacy of tezepelumab in adults with severe uncontrolled asthma.

**Table 1:** Functional and clinical parameters at T0, T3, and T6.

	T0	T3	T6
FEV_1_, L, (% pred.)	1.25 (48%)	1.42 (54%)	1.49 (57%)
FEV_1_/FVC, %	58.92	65.11	62.62
PEF, L/s, (% pred.)	2.76 (43%)	3.79 (60%)	3.38 (54%)
MMEF_25–75_, L/s, (% pred.)	0.70 (31%)	0.89 (40%)	0.87 (39%)
ACT	11	17	21
SNOT‐22	83	62	41
VAS	8	6	4

**Figure 3:**
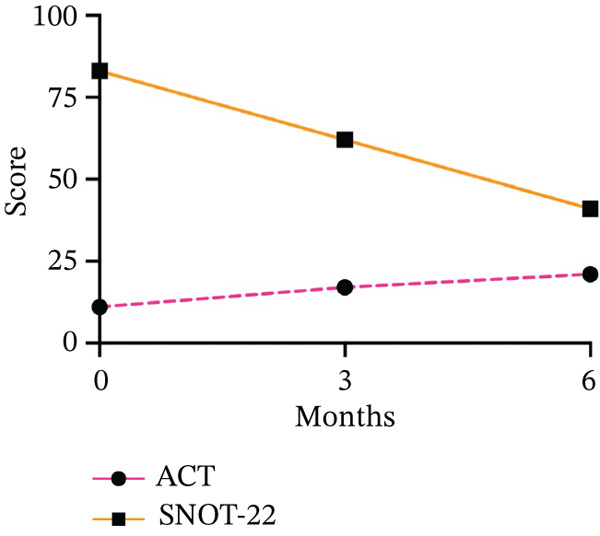
Effects of add‐on biological therapy with tezepelumab on Asthma Control Test (ACT) and Sino‐nasal Outcome Test 22 (SNOT‐22) after 3 and 6 months.

The ACT score increased from 11 to 21 over the 6 months considered in the study, indicating an improvement in the patient′s subjective perception of the disease (Figure 3). This parameter aligned with the literature, particularly in the studies mentioned, PATHWAY and NAVIGATOR, which tested Asthma Control Questionnaire (0ACQ) and AQLQ [3, 5]. ACT appears to be more practical and beneficial in real‐world settings than the ACQ, which is utilized in randomized controlled trials [9, 10].

In our case, the clinical and functional improvement was associated with OCS sparing, which was already evaluated in Phase 3 of a multicenter, randomized, double‐masked, placebo‐controlled study SOURCE [11].

The patient also noted the absence of a need to visit a hospital or medical clinic or to use a reliever when necessary. This reduction in asthma exacerbations was again with the PATHWAY trial [3].

**Figure 4:**
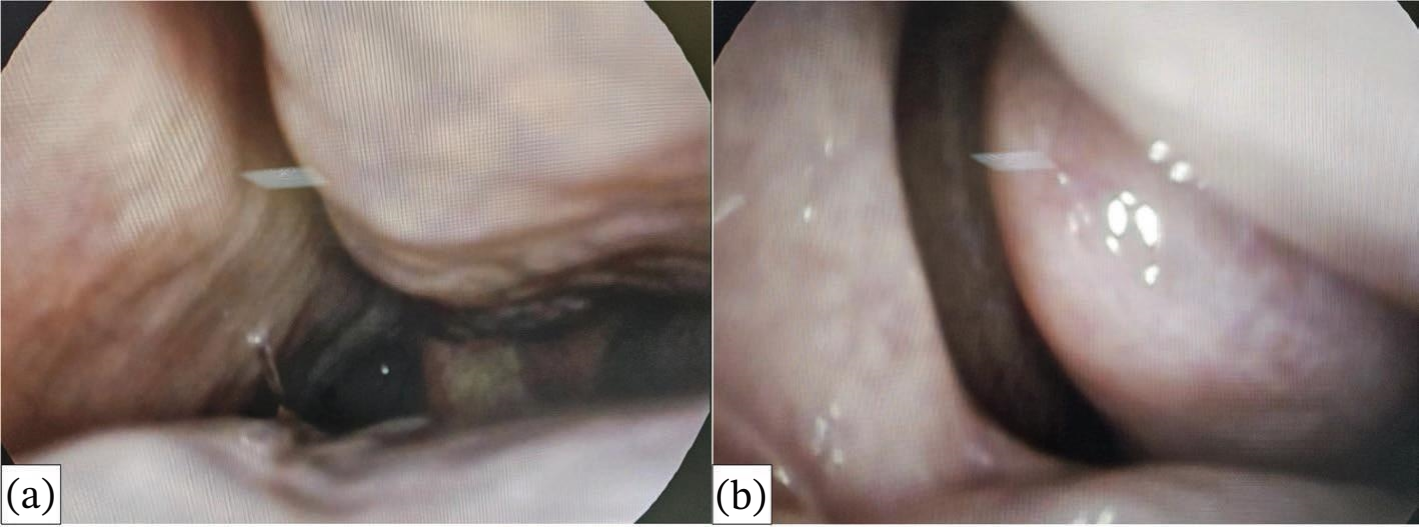
Free left ostiomeatal complex (a) and right ostiomeatal complex (b).

**Figure 5:**
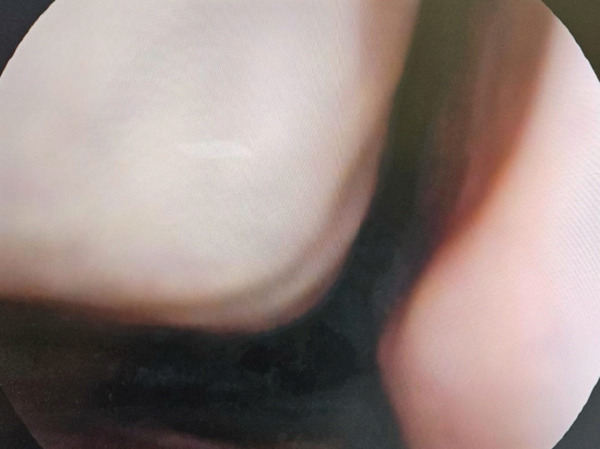
Right nasal cavity, with hypertrophy of the nasal turbinates covered by pale mucosa.

Finally, we performed a rhinoscopy at T6 (Figures 4 and 5). We were aware that the patient had a clinical history of chronic rhinosinusitis with nasal polyps. During the rhinoscopy, we observed hypertrophy of the nasal turbinates, serous endonasal secretion, and a free OMC without nasal polyps. The SNOT‐22 decreased from 83 to 41, indicating moderate nasal obstruction symptoms (Figure 3). Tezepelumab demonstrates efficacy in the treatment of nasal polyposis by inhibiting TSLP, an epithelial cytokine known to provoke Type 2 inflammation. Inhibiting TSLP effectively impedes the activity of immune cells responsible for eosinophilic inflammation, mucus secretion, and tissue edema, thereby decelerating the development of polyps. This upstream approach enables assistance for a diverse range of patients, extending beyond those solely affected by classic Type 2 inflammation. For future investigations, we aim to verify the improvement of nasal polyposis in patients eligible for this biological drug, as recently demonstrated by the Phase III WAYPOINT trial. Previously, a post hoc analysis of the PATHWAY study showed that tezepelumab decreased the exacerbation rate to a greater extent in patients with nasal polyps than in those without nasal polyps [12]. The WAYPOINT study is aimed at assessing the safety and effectiveness of tezepelumab in treating individuals with severe CRSwNP [8]. The trial′s coprimary endpoints were the change from baseline in the endoscopic total nasal polyp score, which measured the size of all nasal polyps, and the participant‐reported nasal congestion score, which was assessed as part of the daily Nasal Polyposis Symptom Diary and measured the biweekly mean nasal congestion. Secondary endpoints included: loss of smell; SNOT‐22 score and Lund–Mackay score; time to surgery decision and systemic corticosteroids for nasal polyposis; time to nasal polyposis surgery decision; and time to systemic corticosteroids for nasal polyposis [8]. Moreover, Cristallo et al. observed effects similar to ours in a 73‐year‐old Caucasian woman with rhinoconjunctivitis, chronic sinusitis and uncontrolled asthma [13].

## 4. Conclusion

Monoclonal antibody treatments offer a better and safer alternative for patients experiencing exacerbations while using high‐dose anti‐inflammatory drugs. However, since current biologics can only block certain Type 2 pathways and not all exacerbations, a significant burden of illness remains. As partial inhibition of Type 2 pathways may not suffice in preventing exacerbations for some individuals, the limitations of existing biologics might arise from inhibiting pathways downstream in the immune cascade while leaving others active. Blocking alarmins, upstream mediators that coordinate broad inflammatory effects and are triggered early in the inflammatory response, presents a viable alternative method that could be effective for a broader range of patients [14].

Several randomized controlled trials have shown a significant therapeutic impact of this molecule. Nonetheless, we assert that the current case report has supplementary value. In fact, we demonstrate that tezepelumab, which blocks TSLP from binding to its receptor, was successful in a real‐world context as an adjuvant biological treatment for severe asthma and nasal polyposis. The patient had previously failed various biologic treatments (mepolizumab, benralizumab, and dupilumab), making her response to tezepelumab clinically significant in a real‐world context. Furthermore, our analysis delineates enhancements in functionality, clinical outcomes, and quality of life over a 6‐month period, including spirometry, ACT, SNOT‐22, and rhinoscopic assessment, so offering longitudinal real‐world data. Moreover, we emphasize the fast response to tezepelumab, with significant enhancements seen within 3 months, pertinent to practical practice.

Therefore, this finding suggests the potential for practical research focused on the effectiveness of this novel medication in various asthma comorbidities.

## Funding

This study was supported by Open access publishing facilitated by Universita degli Studi Magna Graecia di Catanzaro.

## Consent

The patient allowed personal data processing and written informed consent was obtained from the individual participant included in the study.

## Conflicts of Interest

The authors declare no conflicts of interest.

## Data Availability

The data that support the findings of this study are available from the corresponding author upon reasonable request.

## References

[1] Global Initiative for Asthma , Global Strategy for Asthma Management and Prevention, 2023, https://ginasthma.org/.

[2] Gauvreau G. M. , O′Byrne P. M. , Boulet L. P. , Wang Y. , Cockcroft D. , Bigler J. , FitzGerald J. , Boedigheimer M. , Davis B. E. , Dias C. , Gorski K. S. , Smith L. , Bautista E. , Comeau M. R. , Leigh R. , and Parnes J. R. , Effects of an Anti-TSLP Antibody on Allergen-Induced Asthmatic Responses, New England Journal of Medicine. (2014) 370, no. 22, 2102–2110, 10.1056/NEJMoa1402895, 2-s2.0-84901759301, 24846652. 24846652

[3] Corren J. , Parnes J. R. , Wang L. , Mo M. , Roseti S. , Griffiths J. , and van der Merwe R. , Tezepelumab in Adults With Uncontrolled Asthma, New England Journal of Medicine. (2019) 380, no. 21, 10.1056/NEJMoa1704064, 2-s2.0-85029155646. 28877011

[4] Sverrild A. , Hansen S. , Hvidtfeldt M. , Clausson C. M. , Cozzolino O. , Cerps S. , Uller L. , Backer V. , Erjefält J. , and Porsbjerg C. , The Effect of Tezepelumab on Airway Hyperresponsiveness to Mannitol in Asthma (UPSTREAM), European Respiratory Journal. (2021) 59, no. 1, 2101296, 10.1183/13993003.01296-2021. 34049943

[5] Menzies-Gow A. , Colice G. , Griffiths J. M. , Almqvist G. , Ponnarambil S. , Kaur P. , Ruberto G. , Bowen K. , Hellqvist Å. , Mo M. , and Garcia Gil E. , NAVIGATOR: A Phase 3 Multicentre, Randomized, Double-Blind, Placebo-Controlled, Parallel-Group Trial to Evaluate the Efficacy and Safety of Tezepelumab in Adults and Adolescents With Severe, Uncontrolled Asthma, Respiratory Research. (2020) 21, no. 1, 10.1186/s12931-020-01526-6, 33050934. PMC7550847 33050934

[6] Menzies-Gow A. , Ponnarambil S. , Downie J. , Bowen K. , Hellqvist Å. , and Colice G. , DESTINATION: A Phase 3, Multicentre, Randomized, Double-Blind, Placebo-Controlled, Parallel-Group Trial to Evaluate the Long-Term Safety and Tolerability of Tezepelumab in Adults and Adolescents With Severe, Uncontrolled Asthma, Respiratory Research. (2020) 21, no. 1, 10.1186/s12931-020-01541-7, 33087119. PMC7576983 33087119

[7] Emson C. , Diver S. , Chachi L. , Megally A. , Small C. , Downie J. , Parnes J. R. , Bowen K. , Colice G. , and Brightling C. E. , CASCADE: A Phase 2, Randomized, Double-Blind, Placebo-Controlled, Parallel-Group Trial to Evaluate the Effect of Tezepelumab on Airway Inflammation in Patients With Uncontrolled Asthma, Respiratory Research. (2020) 21, no. 1, 10.1186/s12931-020-01513-x, 33050900. PMC7550845 33050900

[8] Lipworth B. J. , Han J. K. , Desrosiers M. , Hopkins C. , Lee S. E. , Mullol J. , Pfaar O. , Li T. , Chen C. , Almqvist G. , Margolis M. K. , McLaren J. , Jagadeesh S. , MacKay J. , Megally A. , Hellqvist Å. , Mankad V. S. , Bahadori L. , and Ponnarambil S. S. , Tezepelumab in Adults With Severe Chronic Rhinosinusitis with Nasal Polyps, New England Journal of Medicine. (2025) 392, no. 12, 1178–1188, 10.1056/NEJMoa2414482, 40106374. 40106374

[9] Jia C. E. , Zhang H. P. , Lv Y. , Jiang R. L. Y. Q. , Powell H. , Fu J. J. , Wang L. , Gibson P. G. , and Wang G. , The Asthma Control Test and Asthma Control Questionnaire for Assessing Asthma Control: Systematic Review and Meta-Analysis, Journal of Allergy and Clinical Immunology. (2013) 131, no. 3, 695–703, 10.1016/j.jaci.2012.08.023, 2-s2.0-84875229895. 23058645

[10] Pelaia C. , Busceti M. T. , Solinas S. , Terracciano R. , and Pelaia G. , Real-life Evaluation of the Clinical, Functional, and hematological Effects of Mepolizumab in Patients with Severe Eosinophilic Asthma: Results of a Single-Centre Observational Study, Pulmonary Pharmacology & Therapeutics. (2018) 53, 1–5, 10.1016/j.pupt.2018.09.006, 2-s2.0-85053478318, 30217438. 30217438

[11] Wechsler M. E. , Menzies-Gow A. , Brightling C. E. , Kuna P. , Korn S. , Welte T. , Griffiths J. M. , Sałapa K. , Hellqvist Å. , Almqvist G. , Lal H. , Kaur P. , Skärby T. , and Colice G. , Evaluation of the Oral Corticosteroid-Sparing Effect of Tezepelumab in Adults With Oral Corticosteroid-Dependent Asthma (SOURCE): A Randomised, Placebo-Controlled, Phase 3 Study, Lancet Respiratory Medicine. (2022) 10, no. 7, 650–660, 10.1016/S2213-2600(21)00537-3, 35364018. 35364018

[12] Emson C. , Corren J. , Sałapa K. , Hellqvist Å. , Parnes J. R. , and Colice G. , Efficacy of Tezepelumab in Patients with Severe, Uncontrolled Asthma With and Without Nasal Polyposis: A Post Hoc Analysis of the Phase 2b PATHWAY Study, Journal of Asthma and Allergy. (2021) 14, 91–99, 10.2147/JAA.S288260, 33568920. 33568920 PMC7868291

[13] Cristallo M. , Filieri M. , Spataro F. , Muolo L. , Nettis E. , and Di Girolamo A. , Successful Treatment of Chronic Rhinosinusitis With Nasal Polyps (CRSwNP) With Tezepelumab: A Case Report, Immunology. Published. (2025) 10.1111/imm.13915, 40024624. 40024624

[14] Oka A. , Klingler A. I. , Kidoguchi M. , Poposki J. A. , Suh L. A. , Bai J. , Stevens W. W. , Peters A. T. , Grammer L. C. , Welch K. C. , Smith S. S. , Conley D. B. , Schleimer R. P. , Kern R. C. , Tan B. K. , Fujieda S. , Okano M. , and Kato A. , Tezepelumab Inhibits Highly Functional Truncated Thymic Stromal Lymphopoietin in Chronic Rhinosinusitis, Journal of Allergy and Clinical Immunology. (2025) 156, no. 2, 463–467.e2, 10.1016/j.jaci.2025.02.031, 40057283. 40057283 PMC12331434

